# Expression of CD34 and α-SMA Markers in Oral Squamous Cell Carcinoma Differentiation. A Histological and Histo-Chemical Study

**DOI:** 10.3390/ijerph18010192

**Published:** 2020-12-29

**Authors:** Afsheen Maqsood, Anwar Ali, Zareena Zaffar, Sameer Mokeem, Sara S. Mokeem, Naseer Ahmed, Nawwaf Al-Hamoudi, Fahim Vohra, Fawad Javed, Tariq Abduljabbar

**Affiliations:** 1 Department of Oral Pathology, Altamash Institute of Dental Medicine, Karachi 75500, Pakistan; afsheenmaqsood@gmail.com; 2 Department of Oral Surgery, Dr Ishrat Ul Ebad Khan Institute of Oral Health Sciences, Dow University, Karachi 74200, Pakistan; anwar.ali@duhs.edu.pk (A.A.); zareena.zj@gmail.com (Z.Z.); 3 Department of Periodontics and Community Dentistry, College of Dentistry, King Saud University, Riyadh 11451, Saudi Arabia; smokeem@ksu.edu.sa (S.M.); nalhamoudi@ksu.edu.sa (N.A.-H.); 4 College of Dentistry, Riyadh Elm University, Riyadh 12611, Saudi Arabia; sararmokeem@gmail.com; 5 Department of Prosthodontics, Altamash Institute of Dental Medicine, Karachi 75500, Pakistan; naprosthodontist@gmail.com; 6 Department of Prosthetic Dental Science, Research Chair for Biological Research in Dental Health, College of Dentistry, King Saud University, Riyadh 11545, Saudi Arabia; fvohra@ksu.edu.sa; 7 Department of Orthodontics and Dentofacial Orthopedics, Eastman Institute for Oral Health, University of Rochester, Rochester, NY 14620, USA; fawjav@gmail.com

**Keywords:** oral squamous cell carcinoma, CD34, α-SMA, myofibroblasts, tumor markers

## Abstract

To reduce morbidity and mortality rates of OSCC cases, early diagnosis, assessment of behavior and prognostic estimates are vital. This study analyzed the expression of CD34 and alpha smooth muscle actin (α-SMA) in OSCC, to establish their significance in diagnosis and prognosis. Primary cases of OSCC, diagnosed with excisional biopsy at multiple cancer treatment centers, were included. Tissue sections were embedded and stained with H & E for histological differentiation and invasion of tumor vessel. Immunohistochemistry was performed using antibodies against CD34 and α-SMA. The chi-square and Pearson correlation coefficient (r) tests were applied for data analysis. Eighty patients with fifty males (62.5%) and thirty females (37.5%) and mean age of 45 ± 14.1 years were evaluated. Buccal mucosa was the most common site for OSCC lesions [36 (45%)]; 47.5% of lesions were moderately differentiated and 33.8% were well-differentiated lesions. Invasion of tumor vessels was observed in 35% of specimens. A significant association was seen between CD34 expression and histological grading of OSCC (*p* < 0.002). Among all poorly differentiated OSCC specimens, expression of CD 34 was low and α-SMA was high. CD 34 is a critical prognostic factor in OSCC diagnosis and increased α-SMA-positive myofibroblasts may indicate aggressive OSCC behavior.

## 1. Introduction

The most frequently occurring cancer in the head and neck region is the oral squamous cell carcinoma (OSCC) originating from the oral keratinocytes, which ranks amongst the 10th most common cancers worldwide [1,2]. About one-fifth (21%) of the cancers in males and about one-tenth (11%) in females are head and neck squamous cell carcinoma (SCC) [3]. Head and neck cancers most commonly occur in the developing world [4]. In South Asia, oral carcinoma is the second most common malignancy among males and females [5]. One of the major global risk factors for carcinoma is smokeless tobacco and betel nut, which is consumed by 20% of the world population. The major role in gross and aggressive behavior of various pathological grades of OSCC is laid by tumoral stroma. Stromal reactions in cancer have a vital diagnostic and prognostic significance [6]. The local aggregation of connective tissue cells and extracellular matrix characterizes epithelial tumors; this phenomenon has been labelled as the stromal reaction. Cells of the stroma give the capability to partake actively in the progression of tumor by secreting proteolytic enzymes, which in turn allow for invasion and metastasis [7].

CD34 is a cluster of differentiation molecule that is seen on several cells inside the body. It is a glycoprotein that is present on the surface of the cell, which works as a cell-cell adhesion factor [8,9,10]. Cells that express CD34 are endothelial cells of blood vessels excluding lymphatics (except pleural lymphatics), mast cells, a sub-population of dendritic cells (which are factor XIIIa negative) in the interstitium and around the adnexa of the dermis of the skin. Studies showcased that normal mucosal stroma and squamous intraepithelial lesions (SILs) enclosed CD34-positive cells that were scattered, but there was an absence of alpha smooth muscle actin (α-SMA)-positive myofibroblasts. On the contrary, the stromal cells of SCC showed α-SMA-positive myofibroblasts. The appearance of α-SMA-positive myofibroblasts and the disappearance of CD34-positive stromal cells are linked with the conversion of squamous intraepithelial lesions (SILs) to SCC. Complete disappearance of CD34-positive cells in the stroma in SCC indicates that they could have been converted into myofibroblasts. The appearance of CD34-positive endothelial cells plays a vital role in understanding the process of angiogenesis in oral cancer and pre-cancer [11].

Alpha-smooth muscle actin (α-SMA) antibody labels smooth muscle cells, myofibroblasts (MF) and myoepithelial cells [12]. For the identification of myofibroblasts, the commonly used marker is smooth muscle actin, which also helps in monitoring its behavior. Increase in the stromal MF is evaluated by the immunoreactivity of α-SMA in OSCC and has been linked with poor prognosis [13]. The expression of alpha smooth muscle actin in the stroma of fibroblasts could be used in the index of OSCC in its supplemental diagnosis and judgment of severity [14]. It is well acknowledged that the activity that is coordinated between epithelial cells and their stroma is central in regulating growth and differentiation in normal as well as pathological conditions. Through the progression of tumor, the stroma imitates disturbed interactions between the neoplastic population and its surroundings. Successful immunotherapy against cancer should essentially include complementary treatments against these tumor-associated fibroblasts. Although CD34 and α-SMA levels among OSCC patients has been reported before, the correlation of these biomarkers in relation to the clinical progression and grading of OSCC is limited. It is therefore hypothesized that CD34 and α-SMA will show association with OSCC differentiation.

Therefore, the purpose of our investigation is to evaluate the expression of CD34 and α-SMA in oral squamous cell carcinoma to confirm their diagnostic and prognostic significance and association with tumor differentiation.

## 2. Materials and Methods

### 2.1. Ethical Considerations

The revised guidelines as documented by the declaration of Helsinki (2013) associated with the experiments in human patients were observed in the study. Participation was voluntary and participants read and completed a written consent form in English. All participants were informed that there were no consequences on withdrawal from the study. The ethics and review board of the Institute of Dental Science provided ethical review and approval for this investigation (Ref No. AIDM/EC/05/2019/07). The sample size for the present study was attained by using the sample size calculation software Open Epi, based on the prevalence of OSCC. With a power of 80% and a confidence level of 95%, a total of 80 patients were selected through (non-probability) quota sampling.

### 2.2. Study Patients and Selection Criteria

In this cross sectional clinical study the samples were selected from patients presenting with oral squamous cell carcinoma. Samples were collected at the Institute of Dental Medicine, Institute of Oral Health Sciences, Diagnostic Lab and Hospital ENT Department. Duration of the study was one year, from November 2018 to November 2019. Only primary cases of OSCC, which were clinically diagnosed with excisional biopsy and neck resections, of all ages in both genders, were included in the study. Smokers, patients with lichen planus, patients with inconclusive biopsy outcomes and hemorrhagic and/or necrotic areas in the specimen were excluded. All the samples obtained were assessed using routine hematoxylin and eosin staining for histological parameters and immunohistochemistry for CD34 and alpha smooth muscle actin.

A 10% neutral buffered formalin solution was used to fix the tissue specimens’ overnight, and gross cut ups using standard protocols were performed. For the next 12 h, these specimens were processed in an automated “Medite TPC 15” tissue processor.

### 2.3. Hematoxylin and Eosin Staining

The tissue sections were subjected to routine embedding, processing and staining with hematoxylin and eosin for histological diagnosis. Hematoxylin staining was performed for a minimum of 90 s with agitation. Slides were rinsed with water (10 dips) until the gross stain was removed and blotted remaining water on a gauze pad. To preferentially remove hematoxylin from non-nuclear components, the slides were dipped three times in acid alcohol (1% HCL in distilled water). Slides were exposed to ammonia water (2% sodium borate) to restore the basic pH of the dye, enhance staining and change the nuclei color from purple to blue. Staining with eosin was performed for 20–30 s, turning the cytoplasm and other constituent’s pink to red. To ensure removal of excess eosin as well as water from the tissues, dehydration of the slide in successively increasing concentrations of alcohol was performed. This was followed by xylene treatment until the cover slipped to avoid drying artifact, which can make interpretation impossible. Excess xylene was removed and the cover slip was mounted using mounting medium. The histological grade was determined according to the degree of differentiation of the tumor (Broder’s classification) [15].

### 2.4. Immunostaining

Immunohistochemistry was performed with a sensitive peroxidase-streptavidin routine using antibodies against CD34 and α-SMA. Slides coated with Histogrip were used for mounting 3–4 µm thick tissue sections embedded in paraffin, which were then dried at 56 °C for 30 min. The tissue sections were deparaffinized using xylene, which was rehydrated in, serialized gradation (100%, 90%, 70% and 50%) using a water–ethanol solution followed by rinsing with deionized water. Retrieval of antigen was performed with target retrieval solution (DAKO, Denmark). A coupling jar was filled with adequate amount of target solution and positioned in the water bath, which was heated up to 95–99 °C. The tissue sections were submerged into a preheated target retrieval solution in water bath and incubated for 20–40 min followed by 20 min of cooling at room temperature. The methodology was partly adapted from a previous study [11].

Once antigen recovery was performed, endogenous peroxidase activity was prevented by immersing slides in peroxidase block solution (3% hydrogen peroxide containing sodium azide) for ten minutes. Following cleaning by TBST (Tris buffer saline with Tween 20, Sigma Aldrich, Kempton Park, South Africa), tissue slides were incubated with the principal antibody of preference, maintaining the principal antibody in optimum dilution for one hour at room temperature. Tissue sections were cleaned to eliminate extra buffer and incubated with peroxidase labeled polymer complex (provided in the Envision system) for 35 min. The tissue sections were cleaned with 1 x TBST buffer solution and distilled water. In addition, antigen antibody color advance was conceded out by applying substrate-chromogen solution. Sections were developed with DAB (3, 4, 3′, 4′- tetra amino biphenyl hydrochloride) for five minutes at 37 °C, cleaned meticulously with distilled water and counter stained with Harris Hematoxylin. The tissue sections were irrigated with running water for ten minutes, and decolorized in 1% acid alcohol. Specimens were dried out in 70%, 80%, 95% and 100% alcohol simultaneously for two minutes and were cleaned in a solution of xylene:phenol (1:1), with two shifts of xylene for two minutes individually and finally fixed in DPX. In support of quality management, one positive control was run with every slide of immune stained sections. Positive controls for antibody were taken from the appendix for CD34 and ASMA [16].

### 2.5. Immunohistochemical Analysis

The methodology was partly adopted from a previous study [17]. The evaluation was conceded out at the point of endothelial cells lining the blood vessels and stromal cells by their membranous staining. CD34 immuno-staining was considered positive when the membranous staining in the endothelial cells of the tumor stroma was stained. α-SMA immuno-staining was considered positive when the cytoplasmic staining in the stromal cells was stained. A quantitative scoring method was performed by counting the number of positive cells and the intensity of staining performed. When the number of positive cells was less than 10%, it was considered as 0 and marked as negative, 10–25% positive cells were evaluated as 1, 25–50% positive cells as 2, 50–75% positive cells as 3, and more than 75% positive cells as 4. When the intensity of staining was evaluated, a weak score was indicated by 1, modest by 2 and strong immuno-reactivity by 3. Two researchers who were blinded to the follow-up independently investigated the immunohistochemical assessment. Consensus was obtained by discussing the cases that had different scores [17].

### 2.6. Statistical Analysis

Statistical analysis was performed using statistical program for social sciences (SPSS- version 25 software, IBM Corporation, Armonk, NY, USA). To investigate relationships that were significant statistically among CD34 and α-SMA expression, the chi-square test was applied. Pearson correlation coefficient (r) and the significance (*p* < 0.05) were calculated. A *p* value of less than 0.05 was considered as statistically significant.

## 3. Results

### 3.1. General Characteristics of the Study Population

A total of 80 patients that suffered from head and neck cancer were selected for this research, out of which 50 (62.5%) were males and 30 (37.5%) were females, showing a male predominance. The mean age of patients was 45 ± 14.1. Buccal mucosa was the most common site. Out of the 80 cases, 36 (45%) were on buccal mucosa, 18 (22.5%) on mandibular buccal mucosa, 2 (2.5%) on maxillary buccal mucosa, 2 (2.5%) on lip (maxilla) buccal mucosa, 12 (15%) on the tongue, 4 (5%) on lips and 4 (5%) on the angle of the mouth (Table 1).

### 3.2. Histopathology

On histological assessment, it was observed that the tumor was well differentiated in 27 (33.8%) patients, moderately differentiated in 30 (37.5%) subjects and poorly differentiated in 23 (28.75%) patients. Moderately differentiated squamous cell carcinoma remained the most commonly encountered, in both males and females (Table 1). Grading in relation to age groups is specified in Table 2, however, the cross tabulation of grading with tumor site is shown in Table 3. Invasion of tumor vessel was seen in 28 (35%) and absent in 52 (65%) cases, however it did not show a significant relation with CD34 expression levels. The demographic and clinical pathologic findings are presented in Table 4.

### 3.3. Immunostaining

**Figure 1:**
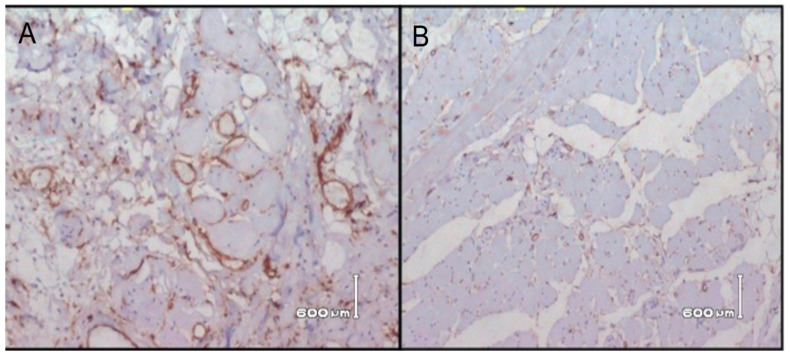
Photomicrograph of Immunohistochemical staining for OSCC, illustrating high (**A**) and low expression (**B**) of CD34 in stromal cells (brown staining).

**Figure 2:**
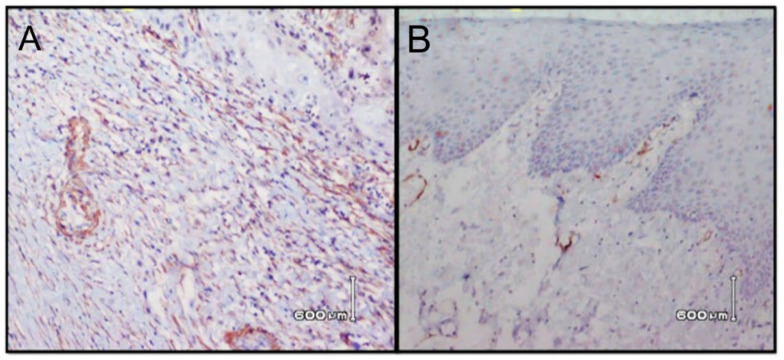
Photomicrograph of Immunohistochemical staining for OSCC, illustrating high (**A**) and low expression (**B**) of α-SMA. α-SMA positive myofibroblasts are observed through brown staining.

The histological grading of OSCC in relation to CD34 and α-SMA is shown in Table 5 and Table 6. Through immunochemistry staining, all 80 specimens were successfully assessed. Stromal positive cells of CD34 staining (Figure 1) were high in seven specimens (8.8%), and low in 73 (91.2%) specimens. α-SMA positive myofibroblast cell staining (Figure 2) was high in 48 specimens (60%), medium in 26 (32.5%) specimens, and low in 6 (7.5%) specimens. A statistically significant correlation was observed between expression of CD34 (*r* = 0.731), (*p* = 0.002) and α -SMA (*r* = 0.823) (*p* = 0.01) and histological grading. Moreover, there was no significant correlation of CD34 with age (*r* = 0.123) (*p* = 0.277) and gender (*r* = 0.043) (*p* = 0.702). Similarly, no significant correlation was found between α -SMA and age (*r* = 0.125) (*p* = 0.269) and gender (*r* = 0.109) (*p* = 0.336).

## 4. Discussion

The present study aimed to analyze the expression of CD34 and α-SMA in OSCC in relation to the level of differentiation in order to establish their significance in diagnosis and prognosis. It was observed that the immunohistochemical expression of CD34 and α-SMA-positive myofibroblasts showed an association with tumor differentiation levels, which indicates an aggressive behavior of OSCC in their presence. Angiogenesis is a critical process in the development of malignant growth and is induced by a complex range of proteins, involving growth factors and extracellular enzymes. The understanding of the angiogenic process in malignant tumors strongly depends on finding the CD34 marker in blood vessel endothelium [18]. In advanced stages of carcinomas, when having increased capability of peripheral growth, such as oropharyngeal and laryngeal malignancies during the process of stroma maturation, the CD34 positive fiber cells presence is not evident due to its conversion into myofibroblasts with alpha smooth muscle actin (α-SMA) expression. The myofibroblasts interrelate chemokines and cytokines through epithelial and connective cells to cause angiogenesis and local tumor incursion [19]. Considering these abilities, myofibroblasts may be used as an important target, during antitumor therapy [20].

In the present study, the most common site of oral cancer was the buccal mucosa (45%). In addition, on histological examination, most excisional biopsies showed moderately differentiated OSCC (37.5%) in this study. Similar rates of 55.9% buccal mucosa involvement were reported in a previous study [21]. The higher prevalence rate of buccal mucosa in the studied population was due to common usage of tobacco, betal quid and areca nut in the form of buccal deposits [22,23]. In a recent study by Mineo et al. [24], high occurrence of distant metastases and increased mortality with high-grade micro-vessel density was associated with CD34. They claimed that there is a considerable association between CD34 expression and tumor metastasis. In the present study, CD34 expression with histological grading of oral squamous cell carcinoma was significant (*p* < 0.05) and an insignificant correlation was noted between CD34 expression and tumor vessel invasion (*p* = 0.23). The disparity in the observed findings is attributed to differences in tumor site. In the study by Minea et al., samples of non-small cell lung cancer were analyzed in contrast to oral cavity lesions in the present study [24].

Nakayama et al. reported that the invasiveness of carcinoma is associated with failure of CD34 marker demonstration in tumor connective tissues and gain of α-SMA active myofibroblasts in the stroma of cancer cells [25]. The majority of the connective tissue cells in the normal epithelium were positive for CD34. On the contrary, the CD34 marker has been found to be negative in the inflamed tissues present around the tumor stroma. A noteworthy relationship was seen in the present study between the compactness of tumor blood vessels in the tissue sample and CD34 expression, which showed disease belligerence. In addition, the present study also showed a significant presence of α-SMA in the stroma of OSCC. This is in line with the findings of Safora et al., which assessed α-SMA in OSCC. Although the sample size in their study was only 54, with a similar mean age of 45 years ± 16 [26].

It is suggested that the stromal response of the host tissue is the most important phase in the development and growth of tumor invasion and CD34 is evident in healthy tissues, however α-SMA was not a feature of normal tissues [27]. It is also reported that α-SMA markers can be helpful in identifying the potential for benign and malignant breast cancer in certain difficult cases [27,28]. This mirrors the unique findings in the present study with all 23 poorly differentiated OSCC cases showing high α-SMA positive levels and low CD34 cell levels. Indicating a strong association of critical diagnostic and prognostic importance.

Therefore, the authors hypothesize that immune-histochemical expression of the angiogenic marker CD34 may prove to be of immense significance as a prognostic factor in the routine diagnosis of OSCC. The stromal ratio amongst the expression of CD34 and α-SMA may cause interest when diagnosing tumors with high malignancy potential. These findings suggest that the detection of a high-risk group of patients is possible with the presence of comparatively increased occurrence of α-SMA, allowing for targeted adjuvant treatments and specific therapies to reduce morbidity and mortality. Moreover, CD34 demonstration is a reliable and consistent technique for gauging tumor vascularity, therefore it is critical to analyze markers including CD34 and α-SMA in early tumor biopsies to estimate the metastatic behavior. However, it is pertinent to mention that the present study was limited due to its cross-sectional nature and did not allow for long-term follow-up of diagnosed patients to assess actual tumor prognosis. Therefore, further randomized controlled trials with long-term follow-ups correlating and confirming the association of prognostic bio-marker (CD-31, CD34 and α-SMA) expression with behavior and prognosis of oral squamous cell carcinomas are recommended.

## 5. Conclusions

The present study strongly indicates that immune-histochemical expression of specific angiogenic markers is of great importance as a prognostic factor in the routine diagnostics of OSCC lesions. In particular, the ratio between stromal expression of CD34 and α-SMA, with increased malignant potential, is of interest. An increase in the amount of α-SMA positive myofibroblasts suggests higher invasive characteristics and weaker prediction of oral squamous cell carcinoma.

## Figures and Tables

**Table 1:** Tumor sites and gender distribution of OSCC lesions among study subjects (*n* = 80).

Tumor Site	Male	Female	Total
Buccal Mucosa	25 (50%)	11 (36.6%)	36 (45%)
Buccal Mucosa with Mandible	13 (26%)	5 (17%)	18 (23%)
Buccal Mucosa with Maxilla	0	2 (7%)	2 (3%)
Buccal Mucosa, Lip with Maxilla	0	2 (7%)	2 (3%)
Tongue	6 (12%)	6 (20%)	12 (15%)
Lip	2 (4%)	2 (7%)	4 (5%)
Angle of Mouth	3 (6%)	1 (3.3%)	4 (5%)
Buccal Mucosa and Lips	1 (2%)	1 (3.3%)	2 (3%)
Total	50 (62.5%)	30 (37.5%)	80

**Table 2:** Oral squamous cell carcinoma grading status with respect to age group (*n* = 80).

Grading	18–28	29–38	39–48	49–58	59–68	69–78	Total
Well differentiated	2	7	8	7	2	1	27
Moderately differentiated	7	5	7	11	6	2	38
Poorly differentiated	2	2	3	5	1	2	15
Total	11	14	18	23	9	5	80

**Table 3:** Oral squamous cell carcinoma differentiation and grading with respect to tumor site (*n* = 80).

Grading	Buccal Mucosa	Buccal Mucosa with Mandible	Buccal Mucosa with Maxilla	Buccal Mucosa, Lip with Maxilla	Tongue	Lip	Angle of Mouth	Buccal Mucosa & Lips	Total
Well differentiated	9	5	0	1	7	1	3	1	27
Moderately differentiated	22	11	0	0	3	2	0	0	38
Poorly differentiated	5	2	2	1	2	1	1	1	15
Total	36	18	2	2	12	4	4	2	80

**Table 4:** Frequencies of clinical–pathologic characteristics among OSCC lesions (*n* = 80).

Categories	No of Patients	Percentage%
Gender		
Male	50	62.5
Female	30	37.5
Age		
≤50	56	70.0
>50	24	30.0
Histological grade		
Well differentiated	27	33.8
Moderately differentiated	30	37.5
Poorly differentiated	23	28.75

**Table 5:** CD34 and Histological Grading of OSCC cross tabulation for association (*n* = 80).

CD34 Positive Cells	Well Differentiated	Moderately Differentiated	Poorly Differentiated	Total	*p* Value
Low	20	30	23	73	0.002
High	7	0	0	7	
Total	27	30	23	80	

**Table 6:** α-SMA and Histological Grading of OSCC cross tabulation for association *n* = 80.

α-SMA Positive Cells	Well Differentiated	Moderately Differentiated	Poorly Differentiated	Total	*p* Value
Low	6	0	0	6	0.01
Medium	14	12	0	26	
High	7	18	23	48	
Total	27	30	23	80	

## Data Availability

The data presented in this study are available in this research article.
